# China’s innovation and research contribution to combating neglected diseases: a secondary analysis of China’s public research data

**DOI:** 10.1186/s41256-023-00288-0

**Published:** 2023-03-14

**Authors:** Jiyan Ma, Lanchao Zhang, Xianzhe Li, Jiashu Shen, Yinuo Sun, Yangmu Huang

**Affiliations:** Department of Global Health, School of Public Health, Peking University, Beijing, China

**Keywords:** Neglected diseases, Research and development, Developing countries

## Abstract

**Background:**

Many emerging and developing economies, such as China, have played the important roles in combating global neglected diseases (NDs). This study aims to explore China’s public landscape of research projects and funding of NDs and to provide empirical evidence on promoting China’s participation in addressing global health priorities that disproportionately affect developing countries.

**Methods:**

We systematically sourced China’s public funding information from the National Natural Science Foundation of China and provincial science and technology agency websites up to August 16, 2019. Following the G-FINDER R&D scope, we screened projects of NDs for analysis. National-funded projects were reviewed on an annual basis for exploring the trends and distribution of funding flows. Information on provincial-funded projects was compared with national projects by disease, research type, and geographical distribution.

**Results:**

A total of 1266 projects were included for analysis and categorized by year, funding source, recipient, disease, research type, region, and province. China’s national public funding for ND research reached a historical peak of USD 16.22 million in 2018. But the proportion of ND research to all public-funded projects was less than 0.5%, and over half of the ND projects were allocated to “*the big three,*” i.e., tuberculosis, HIV/AIDS, and malaria. About 58% of national and provincial ND projects focus on basic research. Economically developed regions and municipalities play dominant roles in leading national ND research, such as Beijing, Shanghai, and Guangdong. Provincial ND projects are primarily driven by endemic regions.

**Conclusions:**

As a new emerging high-tech innovator, China has gradually increased public input to ND-related innovation and research. But there is still a large funding gap among NDs that requires China’s increased support and participation. National development plans and cooperative health needs should be taken into account for China’s participation in promoting global research and development (R&D) for combating NDs.

**Supplementary Information:**

The online version contains supplementary material available at 10.1186/s41256-023-00288-0.

## Background

Neglected diseases (NDs) refer to a variety of infectious diseases that predominantly affect disadvantaged populations with little or no access to health care products. They can lead to life-long deformities, disabilities, and slow deaths while trapping vulnerable people in an unending vicious cycle [1]. The global death toll from NDs reaches more than half a million every year [2]. At least one billion people get chronically infected but lack timely access to affordable treatments [3]. These formidable challenges are set to increase with insufficient water, poor sanitation and hygiene (WASH) infrastructure, and localized pockets of poverty and gender inequality, threatening the well-being of the poorest 1.6 billion people [4].

Research and development (R&D) of new health technologies are essential to sustainably control, eliminate, and eradicate NDs. Compared to neglected tropical diseases (NTDs), the definition of ND weakens geographical characteristics and focuses on those diseases with insufficient existing products due to market failure for product development. Except for “*the big three*” (tuberculosis, HIV/AIDS, and malaria), most NDs are underinvested in the pharmaceutical industry [5]. Since new product development is generally driven by market profitability and attractiveness, the fact that NDs predominantly affect poor people in developing countries leads to a lack of effective and affordable health technologies to prevent, diagnose, and treat these diseases. Globally, only 4.4% of the 850 new products registered between 2000 and 2011 were for NDs [5]. More than 1.7 billion people require treatment for at least one NTD [6]. To reduce disparities in access and delivery of health care, the international community has made significant strides to develop and supply high-quality ND products. The pharmaceutical industry has donated 17 NTD medicines to the WHO and other agencies over the past three decades [7]. However, the medicine donation program alone cannot overcome the ND burden. Long-term R&D commitments from government agencies are needed to support emerging or updated disease targets and access to patients, whether in the form of diagnostics, drugs, or vaccines.

It is also important to consider the characteristics of each ND in prevention and treatment and the unique needs of each country. Many developing countries, particularly LMICs with high ND burdens, are absent from multilateral dialogue and R&D decision-making on disease funding, research type and recipient, and which are the fundamental prerequisites to ending neglect [8]. As the largest developing country and an emerging high-tech innovator, China has demonstrated its responsibility in developing new and affordable health technologies for the global fights against NDs. For example, tribendimidine, developed by Chinese scientists, has proven to be safe and efficacious for treating soil-transmitted *helminth* and *clonorchis sinensis* infections [9]. It has become vitally important for the treatment of parasitic infections that cause a disproportionate burden of diseases in the tropics [10]. Additionally, artemisinin, discovered in traditional Chinese medicine (TCM), became a symbolic breakthrough in tropical medicine research around the world. First-line artemisinin-based combination therapy for *P. falciparum* shows an average efficacy rate of 98.6% in Africa [11]. More than 3.1 billion treatment courses were distributed to malaria endemic regions between 2010 and 2019 [12]. China’s government remains committed to supporting innovation, access, and affordability of schistosomiasis, malaria, HIV, and TB health products in developing countries, even though there are currently no specific policies related to research and innovation for NDs [13]. Previous R&D-related ND studies concentrate on identifying the association between disease burden and health expenditure [14, 15]. Little research has explored the important role emerging and developing economies, such as China, have played in combating global NDs. This study is designed to fill in gaps on China’s national funding of ND research projects. We aim to examine the flow of public funding and the distribution of ND-related scientific and innovation projects in China; identify funding priorities and key patterns and issues; and inform strategies for improving China’s participation in ND-related research and innovation. We believe public information on ND research data would provide valuable insights into the state of China’s progress towards addressing global health priorities and empirical evidence on how China can better participate in global R&D for the treatment of NDs.

## Methods

### Study design

We conducted secondary data analysis research by following the G-FINDER R&D scope (Additional file 1). The G-FINDER survey has annual updates on global investment for ND-related R&D since 2008 [16]. It is the gold standard in sourcing and reporting funding data for products and technologies that disproportionally affect developing countries. The R&D scope defines the matrix of neglected diseases and their relevant products, covering various research activities from basic research, drugs, vaccines, diagnostics, and microbicides to vector control products.

### Searching strategy

Public funding information comes directly from the research projects supported by donor governments. National and provincial data was extracted from the National Natural Science Foundation of China (NSFC) and provincial science and technology agency websites up to August 16, 2019. As China’s primary public research and innovation pillar, the NSFC fund shows national R&D priorities and key ND research issues. Given that local governments design funding strategies in ways that reflect health needs and R&D capacity, we took a closer look at ND-related scientific projects supported by provincial science and technology agencies. The NSFC website shows relatively complete data since 2014, so we choose 2014 as the starting point for this research. For projects that had passed the publicity period on provincial websites, other funding sources (i.e., online press, local news, and public records) were cross-checked for complete project information.

### Inclusion and exclusion criteria

All projects were verified based on the inclusion criteria of the G-FINDER scope. To identify preferred ND projects, we conducted a four-phase systematic review to achieve the highest level of evidence. Records were excluded if they were (i) duplicates, (ii) irrelevant to keywords, (iii) unrelated to NDs or human-transmitted diseases, or (iv) did not meet the G-FINDER scope.

### Data extraction and synthesis

National-funded projects were first reviewed on an annual basis to explore trends and distribution of funding flow. These were then compared with provincial funded-projects to present the absolute and relative shares of public-funded projects by geographical distribution, disease, and research types. For the geographical distribution, this study used the seven geographical regions of China as the basis for analysis following the example of Chen et al. and Ji et al. [17, 18] These seven geographical regions (including northwest, north, northeast, east, central, south, and southwest) were divided based on a strategic zoning approach of history, nationality, economy, technology, and other conditions.

## Results

### Project inclusion process and characteristics

**Fig. 1:**
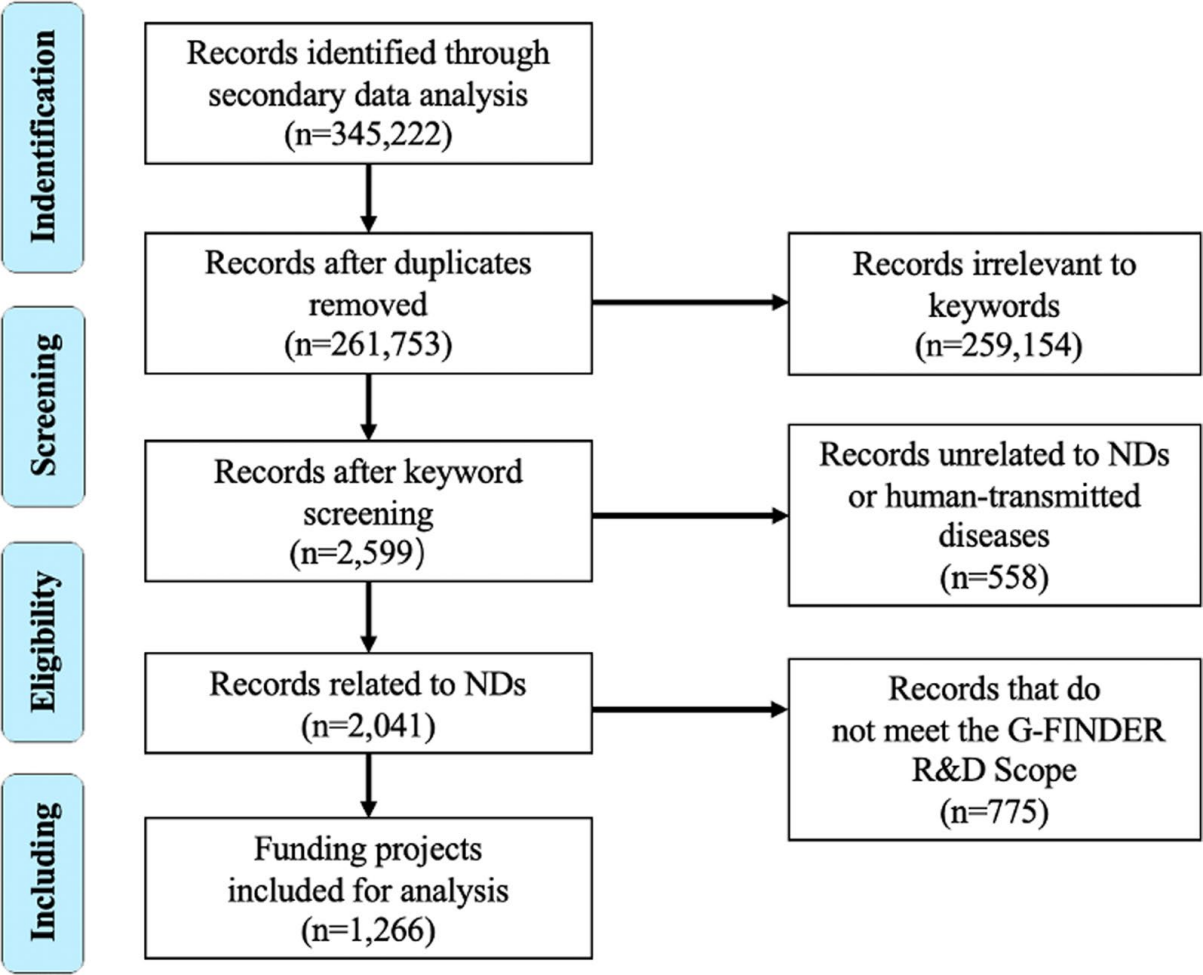
PRISMA flow diagram

**Fig. 2:**
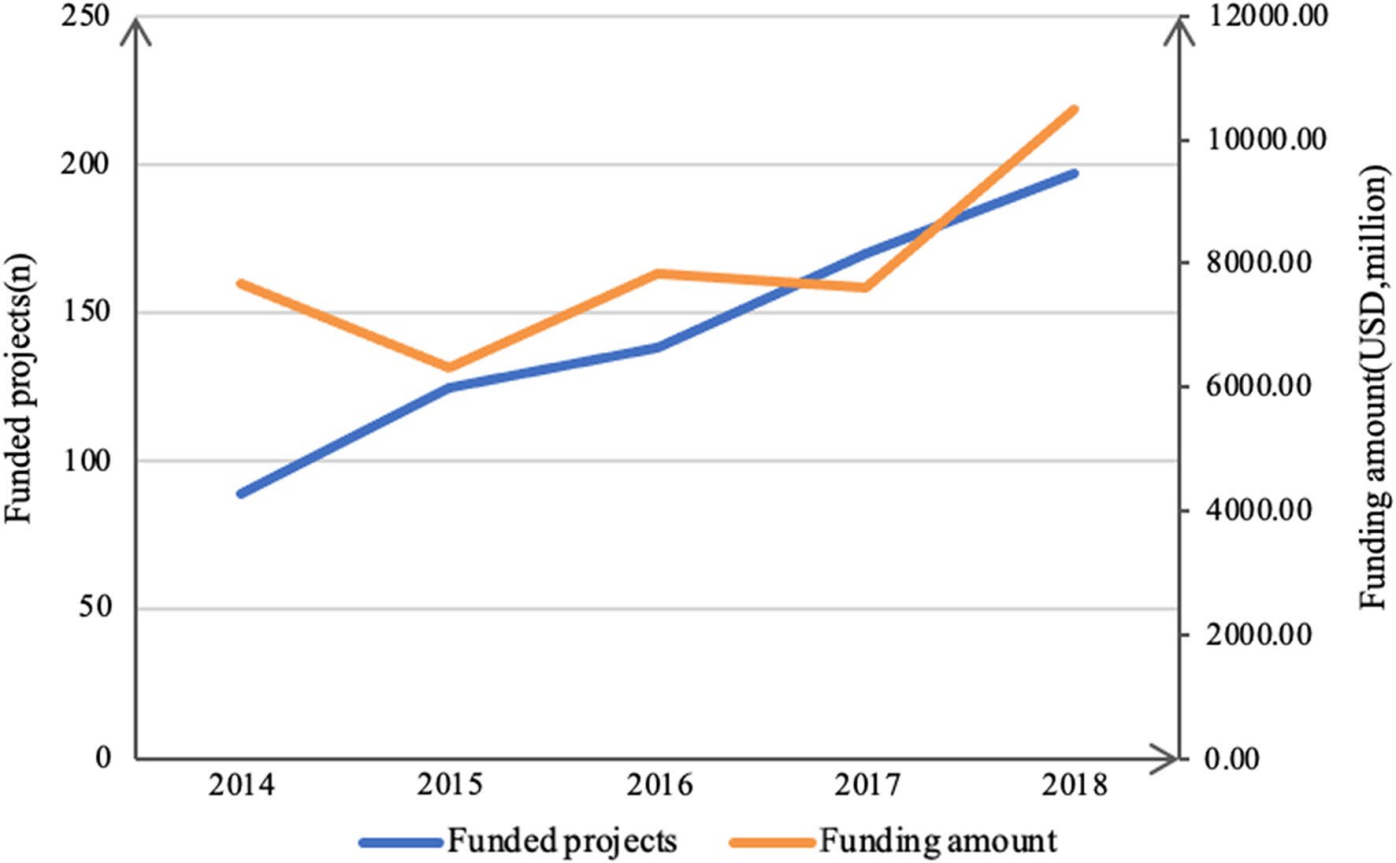
Annual trends of national public-funded projects and funding

A total of 1266 projects were included for analysis and categorized by year, funding source, recipient, disease, research type, region, and province (Fig. 1). Overall, the number of national public-funded projects showed a stepwise increase, from 89 in 2014 to 197 in 2018 (Fig. 2). National public funding slightly decreased in 2015 but increased again from 2016 onwards. By 2018, national public funding of ND-related research rose to its highest point: USD 16.22 million. Funding from 2015 to 2018 doubled. Average funding per project increased from USD 78,160 to USD 82,335 between 2015 and 2018.

### Disease types

**Fig. 3:**
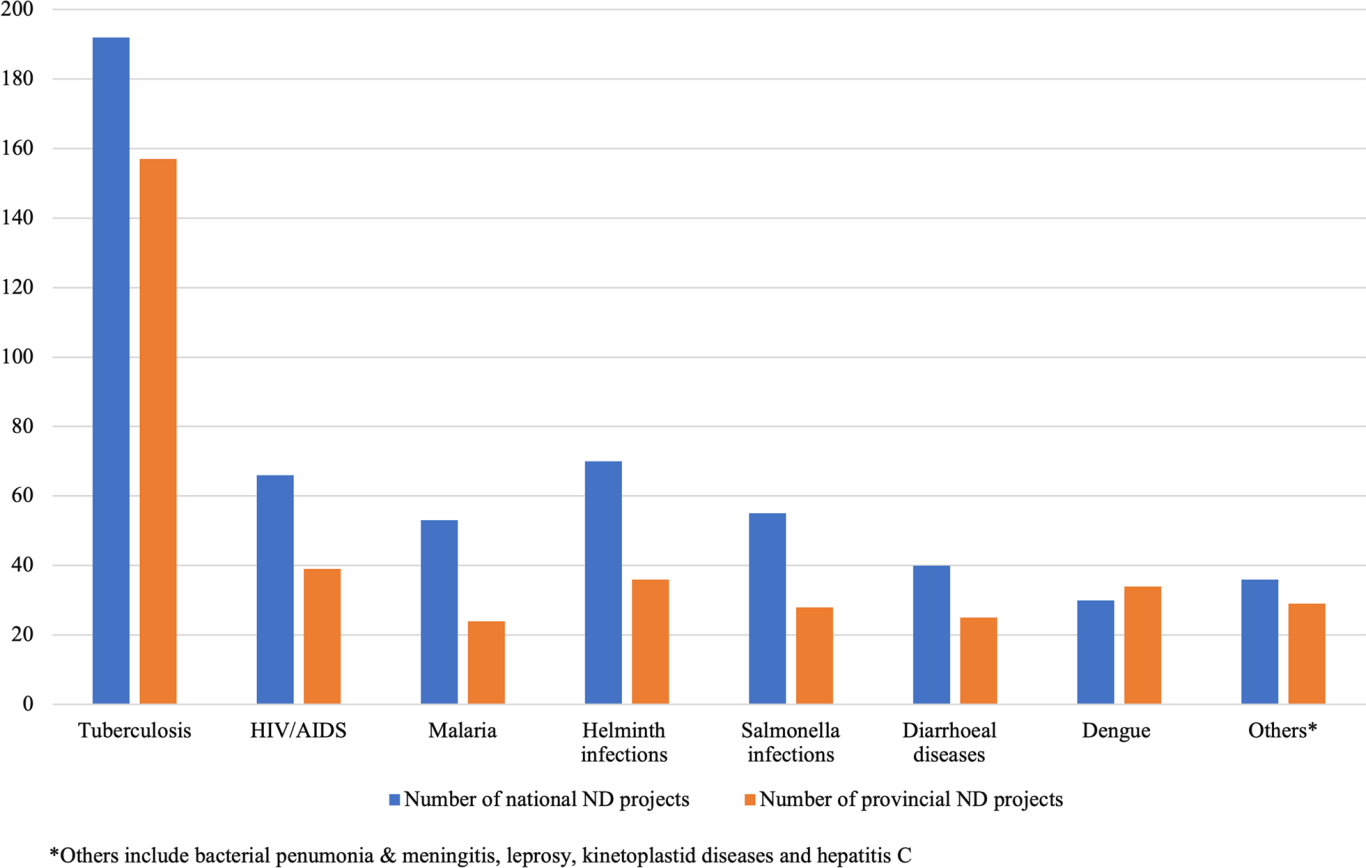
Total number of 2017–2019 national and provincial ND projects by disease type

Among national-funded ND projects, tuberculosis was the key contributor over the 2017–2019 period, with an annual average of 64 projects (Fig. 3). Helminth, HIV/AIDS, and salmonella infections were the highest ranking after tuberculosis, with an average of approximately 23, 18, and 18 projects per year. The NSFC allocated over half of ND projects to “*the big three*” diseases—35.4% to tuberculosis, 12.2% to HIV/AIDS, and 9.8% to malaria.

The total number of ND projects sponsored by provincial public funding was lower than national funding across disease types. Tuberculosis, the highest-funded disease, has an annual average of 52 projects granted by provincial funding, 23.01% lower than the national level (64 projects). Dengue is the only disease with more provincial-funded projects (34 projects) than national projects (30 projects). HIV/AIDS accounts for the second-largest (10.5%, 39 projects) financed by provincial funding, followed by helminth (9.7%, 36 projects) and salmonella (7.5%, 28 projects).

### Research types

**Fig. 4:**
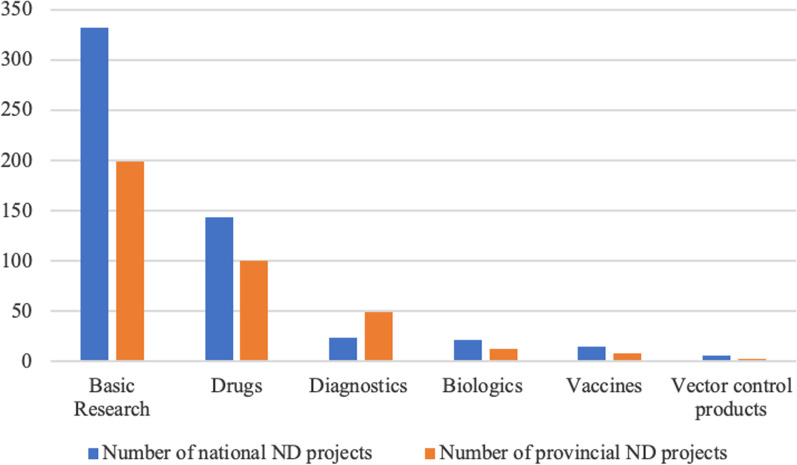
Total number of 2017–2019 national and provincial ND projects by research type

Figure 4 compares national- and provincial-funded projects by research type between 2017 and 2019. Over 80% of national ND projects were deployed to do basic (61.25%, 332 projects) and drug research (26.57%, 144 projects), while other types, including diagnostics, biologics, vaccines, and vector control products share 4.43% (24 projects), 3.87% (21 projects), 2.77% (15 projects), and 1.11% (6 projects) of national projects, respectively. The distribution of provincial funded-projects by research type is broadly similar, except that the number of diagnostic-related provincial projects (49 projects) is about twice that of national projects (24 projects). Vector control products account for the lowest proportion of public-funded projects in terms of research type for both national and provincial levels.

### Geographical distribution

Between 2017 and 2019, a total of 542 projects were funded by national grants focused on ND research and innovation, accounting for 0.42% of NSFC projects across fields (Table 1). Among all national-funded ND projects, the East China region contributed the largest portion (31.37%) with 170 projects. Provinces with the highest proportion of ND projects were Beijing and Shanghai (which were also considered to be centrally-administered municipalities), accounting for 93 (17.16%) and 74 (13.65%) ND projects, respectively. Among all 129,856 NSFC projects funded between 2017 and 2019, the region with the highest ND-related proportion was the South China region. Provinces contributing the largest funds for ND projects were Ningxia (2.65%), Qinghai (1.58%) and Guizhou (1.15%), which also happen to be ND endemic areas. No ND-related NSFC project was observed in Tibet.

**Table 1:** Geographical distribution of national- and provincial-funded projects between 2017 and 2019

Region	Province	National funded projects				Provincial funded projects			
		ND	Of ND’s total (%)	Total	ND Of total (%)	ND	Of ND’s total (%)	Total	ND Of total (%)
Northeast China	Jilin	15	2.77	2171	0.69	8	2.15	2111	0.38
	Liaoning	11	2.03	3882	0.28	6	1.61	1135	0.53
	Heilongjiang	8	1.48	2596	0.31	2	0.54	1211	0.17
	Subtotal	34	6.27	8649	0.39	16	4.30	4457	0.36
North China	Beijing	93	17.16	21,581	0.43	17	4.57	2652	0.64
	Tianjin	6	1.11	3041	0.20	4	1.08	1041	0.38
	Inner Mongolia	5	0.92	872	0.57	0	0.00	105	0.00
	Shanxi	3	0.55	1201	0.25	8	2.15	5840	0.14
	Hebei	3	0.55	1395	0.22	–	–	–	–
	Subtotal	110	20.30	28,090	0.39	29	7.80	9638	0.30
Northwest China	Ningxia	13	2.40	490	2.65	3	0.81	545	0.55
	Shaanxi	11	2.03	6394	0.17	–	–	–	–
	Xinjiang	8	1.48	1193	0.67	21	5.65	2087	1.01
	Qinghai	3	0.55	190	1.58	3	0.81	1269	0.24
	Gansu	2	0.37	1967	0.10	0	0.00	362	0.00
	Subtotal	37	6.83	10,234	0.36	27	7.26	4263	0.63
East China	Shanghai	74	13.65	12,198	0.61	20	5.38	6641	0.30
	Jiangsu	39	7.20	12,520	0.31	11	2.96	2952	0.37
	Shandong	27	4.98	5852	0.46	9	2.42	6169	0.15
	Jiangxi	12	2.21	2621	0.46	1	0.27	2034	0.05
	Zhejiang	11	2.03	6134	0.18	28	7.53	5930	0.47
	Anhui	5	0.92	2039	0.25	8	2.15	4195	0.19
	Fujian	2	0.37	2697	0.07	8	2.15	6761	0.13
	Subtotal	170	31.37	44,061	0.39	85	22.85	34,682	0.25
Central China	Hubei	33	6.09	8971	0.37	12	3.23	3346	0.36
	Henan	10	1.85	2737	0.37	39	10.48	11,113	0.35
	Hunan	3	0.55	2599	0.12	24	6.45	5840	0.41
	Subtotal	46	8.49	14,307	0.32	75	20.16	20,299	0.37
South China	Guangdong	71	13.10	11,138	0.64	50	13.44	9693	0.53
	Guangxi	6	1.11	1744	0.34	7	1.88	1507	0.46
	Hainan	1	0.18	567	0.18	20	5.38	2765	0.72
	Subtotal	78	14.39	13,449	0.58	77	20.70	13,965	0.55
Southwest China	Yunnan	21	3.87	2314	0.91	23	6.18	7588	0.30
	Sichuan	16	2.95	4698	0.34	20	5.38	8110	0.25
	Chongqing	15	2.77	2662	0.56	–	–	–	–
	Guizhou	15	2.77	1302	1.15	13	3.49	2113	0.63
	Tibet	0	0.00	90	0.00	7	1.88	819	0.86
	Subtotal	67	12.36	11,066	0.61	63	16.94	18,630	0.34
Total		542	100.00	129,856	0.42	372	100.00	105,934	0.35

From the provincial perspective, a lower level of public input can be seen in both the total number of ND projects and the proportion of ND to all fields. A total of 372 provincial-funded projects focused on NDs, accounting for 0.35% of the projects of all fields. Among all ND-related projects, the East China region contributes the highest portion (22.85%, 85 projects). Guangdong had the largest number of projects (50), accounting for 13.44% of all ND projects. Among all 105,934 provincial-funded ND projects between 2017 and 2019, the Northwest China region contributed the highest portion. No ND-related research projects were observed in Inner Mongolia and Gansu.

## Discussion

China's public funding for NDs has steadily increased after 2015, but overall, investment is still small. The number of national-funded projects exceed provincial projects. China's investment in NDs is primarily dominated by “*the big three*” and basic research. Economically-developed municipalities play dominant roles in leading national ND research, such as Beijing, Shanghai, and Guangdong. Provincial ND projects are primarily driven by endemic regions.

The patterns of disease and research type in China parallel global R&D funding flow. There is global consensus that new medical technologies—diagnostics, new chemical entities (NCEs), and highly effective vaccines—are desperately needed to control tuberculosis, HIV/AIDS, and malaria. However, diarrheal diseases, salmonella infections, and bacterial pneumonia & meningitis also represent substantial contributions to global burden estimates [19]. Research and Development activities lag far behind “*the big three,*” disproportionate to their importance for global health. Some Chinese patent medicines have shown research progress in relieving chronic diarrhea and antibiotic-resistant bacterial diarrhea [20]. This presents a promising funding opportunity for China to increase R&D support for global ND priorities.

More than half of the R&D funding for ND treatments was in basic research. Yet, without strong public support to translate these discoveries into tools, basic research can only help identify new drug targets, biomarkers, and vector control strategies [21]. For most NDs, such as hookworm, schistosomiasis, and dengue, new molecular entities (NMEs), preventive vaccines, and easy-to-use diagnostics are urgently needed to enhance availability and accessibility of medical products in African and Southeast Asian countries [22]. As China’s domestic NDs reflect international patterns, it could make more substantial contribution to global health by promoting innovation and access to health products for the treatment of NDs.

It is worth noting that the distribution of research projects on specific diseases has geographical characteristics. Most projects are concentrated in economically-developed areas with strong R&D capacities, such as Beijing, Shanghai, and Guangzhou, whereas provincial projects are primarily driven by endemic ND regions, including Ningxia, Qinghai, and Guizhou. The breakdown of funded projects according to research type is not the same for national and provincial projects. This highlights the differences in R&D needs and priorities among central and local governments. Compared to provincial funding, national ND research focuses on basic research and drugs. This may be because the NSFC focuses on key projects that break technical bottlenecks, especially in mainstream national economic and social development, and distributes a larger portion of funding to early research stages. Provincial R&D focuses more on the functional side as in portable diagnostics, which could help strengthen local disease surveillance systems and promote timely access to affordable treatments. This scenario suggests that public funding could be better targeted, especially concerning national development and local health needs.

The COVID-19 pandemic had a double-edged impact on ND research and innovation. On one hand, COVID-19 deprived global efforts and funding for ND research and interfered with the accessibility and availability of ND tools [23]. Most manufacturers were unable to guarantee supply chains during global public health emergencies. On the other hand, the COVID-19 outbreak highlighted the significance of strengthening the capacity of global infectious disease research. The increasing political commitment and funding for emerging infectious diseases could help expand ND talents and resources, especially in developing countries. The COVID-19 pandemic has also raised awareness of public hygiene concepts and healthy behaviors. For example, the WASH interventions are applicable in preventing both COVID-19 and NDs [3].

In the past months, China has made remarkable achievements in tackling the COVID-19 pandemic with significant R&D input. The world has recognized its research contribution to the possible end of NDs. China’s government stated on multiple occasions that it would help developing partners prevent and control NDs with health supplies and resources. However, there are no consistent R&D policies or incentives supporting ND research nationwide. Therefore, the government would need to transform the culture of ND work toward a culture of innovation with sustainable investment and policy attention. Strengthening partnership and collaboration between public and private actors with diversified R&D incentivizing policies upfront could help deploy limited resources to R&D priorities and accelerate the translation of basic research to promising ND products and technologies.

Our study has several limitations. First, even though our study focuses on analyzing China’s public landscape of funding for ND research projects, other sectors (including enterprise, philanthropy, and private) have also participated in promoting ND-related R&D. Those contributions need further analysis in future studies. Second, due to limitations of funding data availability, we were only able to access information disclosed by the NSFC and provincial science and technology agencies. There are some international projects funded by China’s public funding for ND research that haven’t been exposed by public data sources, which means overall public funding for ND R&D in China will be underestimated. Third, the required level of data protection, potential disclosure approaches, and the availability of other external data sources differ across provinces, which could impact the obtained number of funded projects and funding amounts across different provincial science and technology agencies. Notwithstanding, our study aimed to collect all available funding information from national and provincial levels. The overall trends of national public funding of ND research, as well as its comparison with provincial public input, would not be significantly impacted during the same period.

## Conclusions

As a new emerging high-tech innovator, China has gradually increased its public investment in ND-related innovation and research, though overall funding is still limited. There is a large funding gap among NDs that requires China’s increased support and participation. The fact that most projects are concentrated in economically developed regions implies the socioeconomic driving forces behind scientific research in developing countries. Through participating in and promoting global R&D, China should consider national development plans and cooperative health needs. Strengthening partnership and collaboration between public and private actors with diversified upfront R&D incentives could help deploy limited sources to R&D priorities and accelerate the translation of basic research to promising ND products and technologies.

## Supplementary Information

**Additional file 1.** G-Finder R&D Scope.

## Data Availability

The datasets used and/or analysed during the current study are available from the corresponding author on reasonable request.

## References

[1] Molyneux DH, Savioli L, Engels D (2017). Neglected tropical diseases: progress towards addressing the chronic pandemic. Lancet.

[2] Bhutta ZA, Sommerfeld J, Lassi ZS, Salam RA, Das JK (2014). Global burden, distribution, and interventions for infectious diseases of poverty. Infect Dis Poverty.

[3] World Health Organization. Ending the neglect to attain the Sustainable Development Goals: A road map for neglected tropical diseases 2021–2030. [Available from: https://www.who.int/neglected_diseases/resources/who-ucn-ntd-2020.01/en/.

[4] The L (2019). 2020: a crucial year for neglected tropical diseases. Lancet.

[5] Pedrique B, Strub-Wourgaft N, Some C, Olliaro P, Trouiller P, Ford N (2013). The drug and vaccine landscape for neglected diseases (2000–11): a systematic assessment. Lancet Glob Health.

[6] World Health Organization. Neglected tropical diseases: treating more than one billion people for the fifth consecutive year [Available from: https://www.who.int/neglected_diseases/news/treating-more-than-one-billion-people-fifth-consecutive-year/en/.

[7] Bradley M, Taylor R, Jacobson J, Guex M, Hopkins A, Jensen J (2021). Medicine donation programmes supporting the global drive to end the burden of neglected tropical diseases. Trans R Soc Trop Med Hyg.

[8] Yamey G SM, Moran M, Diab MM, McDade KK, Mao W, Chodavadia P, Zimmerman A, HuangY, Chowdhary C, Karanja R, Madikizela M, Ogbuoji O. Developing an aggregator mechanism for late-stage clinical trials of neglected disease product candidates: the center for policy impact in global health. Duke Global Working Paper Series: number 23, October 2020; [Available from: https://centerforpolicyimpact.org/wp-content/uploads/sites/18/2020/10/Aggregator-for-Late-Stage-Trials_Working-Paper-FINAL.pdf.

[9] Qian MB, Yap P, Yang YC, Liang H, Jiang ZH, Li W (2013). Efficacy and safety of tribendimidine against clonorchis sinensis. Clin Infect Dis.

[10] Bergquist R (2016). Tribendimidine: great expectations. Lancet Infect Dis.

[11] World Health Organization. WHO status reports on artemisinin resistance and ACT efficacy [Available from: https://www.who.int/malaria/areas/drug_resistance/updates/en/.

[12] World Health Organization. World malaria report 2020 [Available from: https://www.who.int/publications/i/item/9789240015791.

[13] National Heath Committee. Beijing declaration of the ministerial forum of China-Africa health development 2013 [Available from: http://www.nhc.gov.cn/gjhzs/s3590/201308/da8ad62e487a481f987e631e1318c6fc.shtml.

[14] Gillum LA, Gouveia C, Dorsey ER, Pletcher M, Mathers CD, McCulloch CE (2011). NIH disease funding levels and burden of disease. PLoS One.

[15] Sampat BN, Buterbaugh K, Perl M (2013). New evidence on the allocation of NIH funds across diseases. Milbank Q.

[16] Policy Cures Research. 2019 G-FINDER report: neglected disease research and development [Available from: https://www.policycuresresearch.org/analysis.

[17] Chen W, Zheng R, Baade PD, Zhang S, Zeng H, Bray F (2016). Cancer statistics in China, 2015. CA Cancer J Clin.

[18] Ji HM, Wang J, Meng B, Cao Z, Yang T, Zhi GQ (2022). Research on adaption to air pollution in Chinese cities: Evidence from social media-based health sensing. Environ Res.

[19] Institute for Health Metrics and Evaluation. Global Burden of Disease Study 2017 [Available from: http://ghdx.healthdata.org/gbd-2017/code/cod-8.

[20] Bi CR, Jing W, Xie XF, Liu YJ (2021). Efficacy and mechanism of traditional Chinese medicine in relieving antibiotic-resistant bacterial diarrhea in children: study protocol for a randomized controlled trial. Trials.

[21] Reed SL, McKerrow JH (2018). Why funding for neglected tropical diseases should be a global priority. Clin Infect Dis.

[22] Aerts C, Sunyoto T, Tediosi F, Sicuri E (2017). Are public-private partnerships the solution to tackle neglected tropical diseases? A systematic review of the literature. Health Policy.

[23] Molyneux DH, Aboe A, Isiyaku S, Bush S (2020). COVID-19 and neglected tropical diseases in Africa: impacts, interactions, consequences. Int Health.

