# Supplementary material for: China’s innovation and research contribution to combating neglected diseases: a secondary analysis of China’s public research data
Source: Glob Health Res Policy. 2023 Mar 14;8:6. doi: 10.1186/s41256-023-00288-0 (PMC10010952; doi:10.1186/s41256-023-00288-0)
Supplement: Supplementary file 1 — Additional file 1. G-Finder R&D Scope. [file 41256_2023_288_MOESM1_ESM.docx]

**Appendix 1: List of Neglected Diseases**

**I. SCOPE OF NEGLECTED DISEASE**

*Our study followed the list of neglected diseases set out by the G-FINDER Report, which has been defined by an expert international advisory committee, in line with the following three criteria:*

*1. The disease disproportionately affects people in low- and middle-income countries*

*2. There is a need for new products (i.e., there is no exisiting product, or improved or additional products are needed)*

*3. There is market failure (i.e., there is insufficient commercial makret to attract R&D by private industry)*

**1. LIST OF NEGLECTED DISEASE**

1.1 HIV/AIDS

1.2. Malaria

1.3 Tuberculosis

1.4 Rotavirus

1.5 Shigella

1.6 Cholera

1.7 Cryptosporidiosis

1.8 E. coli

1.9 Leishmaniasis

1.10 Sleeping sickness

1.11 Schistosomiasis

1.12 Tapeworm

1.13 Hookworm

1.14 Strongyloidiasis

1.15 Roundworm

1.16 Multiple helminth infections

1.17 Typhoid and paratyphoid fever

1.18 Non-typhoidal S. enterica

1.19 Multiple Salmonella infections

1.20 Dengue

1.21 S. pneumoniae

1.22 N. meningitidis

1.23 Hepatitis C

1.24 Leprosy

**Appendix 2: Neglected Disease R&D Scope**

**I. BASIC RESEARCH**

*Studies that increase scientific knowledge and understanding about the disease, disease processes, pathogen or vector, but which are not yet directed towards a specific product. Please see section X for disease-specific restrictions to research activities in this category.*

**1. NATURAL HISTORY AND EPIDEMIOLOGY**

**1.1**Basic mechanisms of disease transmission

**1.2**Disease prevalence in relation to human genotype, strain variation, and

inoculation rates

**1.3**Genetic diversity and phylogeny

**1.4**Epidemiological research on the roles of human behaviour and effects of specific host genotypes on disease transmission

**1.5**Epidemiological research on host genetic factors influencing the prevalence of disease (e.g., sickle cell, HLA type, Rh factor) or the impact of disease in select host genotypes

**1.6**Epidemiological research on the distribution of pathogen, vectors and the prevalence of morbidity and mortality due to the disease that is NOT related to the development of a specific product

**1.7**Epidemiological research on antigenic variability; population studies of human immunity to the disease

**1.8**Epidemiology of drug resistance or evolutionary studies on resistance development for established, existing drugs

**1.9**Epidemiological research related to vector behaviour and ecology, and vector control

**2. IMMUNOLOGY OF DISEASE**

**2.1**Defining signalling pathways of immune function (mechanisms of systemic and/or mucosal immunity)

**2.2**Interaction and impact of the signalling pathways with the pathogen

**2.3**Development of assays or tools potentially useful for drug, vaccine, microbicide, or biologic research & development

**2.4**Identification of immune correlates of protection, including *in vivo and in vitro* studies on the protective immune response (cellular, humoral, and/or mucosal)

**2.5**Investigating the immune response to particular antigens; studies of specific antigens or immunogens proposed as vaccine or biologic candidates

**2.6**Development of animal models to determine immune correlates of protection

**2.7**Genetics of the immune response to the disease and effects of antigen polymorphism or genetic diversity on specific vaccine or biologic candidates (as recognised from field studies)

**3. BIOLOGY OF DISEASE**

**3.1**Structure and morphology of different developmental stages

**3.2**Host-parasite interactions and the biology of pathogen interaction with the vector host

**3.3**Biology of invasion of host cells (entry mechanisms)

**3.4**Localisation of pathogen proteins or antigens

**3.5**Development of culture and purification tools to assist in study of the pathogen

**3.6**Descriptions of pathogenic species and characterisation of strains or subtypes in animal models (course of infection, susceptibility of different hosts)

**3.7***In vitro* studies of interactions between the pathogen and other infectious agents (e.g. Epstein-Barr virus)

**4. BIOCHEMISTRY OF THE PATHOGEN**

**4.1**Metabolism and nutrition

**4.2**Protein sequencing, enzymology, and protein and enzyme characterisation (including antigen analysis)

**4.3**Signal transduction; translation, processing and export of proteins

**4.4**Glycosylation, Glycosylphosphatidylinositol (GPI) anchors, transporters, ion channels, mitochondrial metabolism, and electrophysiology studies

**4.5**Influence of the pathogen on host-cell biochemistry

**4.6**Characterisation of antigen/protein diversity of pathogenic strains and subtypes

**4.7**Characterisation of proteins and molecular basis for host-cell invasion

**4.8**Analysis & characterisation of drug-resistant strains and studies probing drug resistance mechanism/s or pathways

**4.9**Non-specific research on the pathogen or host targets to identify potential drug, vaccine, biologic, or diagnostic targets (i.e. target identification)

**5. GENETICS OF THE PATHOGEN**

**5.1**Studies on chromosomes; genomic maps; genetic crosses

**5.2**Cloning and sequencing of genes; cDNAs for functional proteins (including drug targets and vaccine candidates)

**5.3**Expression of proteins from cloned genes; RNA analyses

**5.4**Control and timing of gene expression; post-transcriptional processing

**5.5**Analysis and characterisation of genes involved in drug resistance

**5.6**Genetics of antigenic variability

**5.7**Techniques for the genetic transformation of the pathogen

**5.8**Tests for genotyping the pathogen for laboratory use

**6. BIOINFORMATICS AND PROTEOMICS**

**6.1**Microarray analysis

**6.2**Genome annotation - gene predictions

**6.3**Comparative genomics, sequence alignment, genome assembly

**6.4**Variation, single nucleotide polymorphisms (SNPs)

**6.5**Database applications, data mining tools

**6.6**Structural and functional genomics

**6.7**Structural and functional proteomics

**6.8**Proteome analysis, protein structure alignment

**7. PATHOPHYSIOLOGY AND DISEASE SYMPTOMS**

**7.1**Clinical diagnosis and clinical observations of the disease presentation and pathophysiology in humans and in animals

**7.2**The role of nutritional status in determining disease severity and treatment effectiveness

**7.3**Histopathology of the disease in humans and in animals

**7.4**The mechanisms of pathology of the disease including the role of the host immune system, and expression of adhesion molecules

**7.5**Development of improved animal models to study disease pathophysiology, to evaluate the biological properties of drugs and microbicides

**7.6**Identification of biomarkers for diagnostics or therapeutic monitoring

**7.7**Studies of the mechanisms by which particular susceptible/resistant mammalian host genotypes exert their effect

**7.8**Research on the effects of host co-morbidities and secondary effects of pathogen invasion (e.g., research on anaemia /neurological effects of malaria)

**7.9**Interactions between the disease and other relevant concurrent infections, including determining timing and establishment of infection

**8. VECTOR BIOLOGY, BIOCHEMISTRY, AND GENETICS**

**8.1**Characterisation of vector behaviour and ecology

**8.2**Studies of vector susceptibility to infection; studies of parasites and pathogens of vectors (including potential biological control agents)

**8.3**Identification of genes responsible for disruption of parasite/virus growth, genetic transformation of vectors, and insect transposable elements

**8.4**Target identification of vector sites that may become the subject of *in vitro* screening or molecular design

**8.5**Development of tests for vector identification, taxonomy and systematics, and for the identification of infected vectors

**8.6**Studies evaluating resistance development, including the genetics and transmission of pesticide resistance

**II. DRUGS**

*Research activities and processes necessary to develop and improve new small molecule compounds specifically designed to prevent, cure or treat neglected diseases; including drug discovery or design, preclinical and clinical development and other activities essential for successful drug development and uptake. Please see section X for disease-specific restrictions to research activities in this category.*

**9. DISCOVERY AND PRECLINICAL**

*Research activities targeted at discovering and optimising investigational small molecule compounds including the processes needed to allow new chemical entities to proceed to human trials; including:*

**9.1**Target validation, characterisation, and selection

**9.2**High throughput screening, lead optimisation

**9.3**Development of analytical tests for assaying drugs, including the development of animal models

**9.4**Research on drugs from natural products; identification and characterisation of active ingredient

**9.5**Research on the effects of drug treatment on immune status

**9.6**Measurement of the activity of potential drugs *in vitro* and in animal models; including safety and efficacy studies necessary to satisfy Investigational New Drug (IND) requirements

**9.7**Studies evaluating the activity of new drugs on drug-resistant strains, their effect on genes involved in drug resistance, or their effect on resistance pathways

**9.8**Development of tests for drug susceptibility of the pathogen for research purposes

**9.9**Drug pharmacokinetic, toxicity and metabolism studies *in vitro* and in animal models, including bioavailability, adsorption, metabolism, and excretion (ADME) studies

**9.10**Chemistry and synthesis of drugs, including process and scale-up manufacture, production of Good Laboratory Practice (GLP) and Good Manufacturing Practice (GMP) batch for toxicology studies; and other Chemistry and Manufacture Control (CMC) activities required to allow new chemical entities to proceed to human trials

**9.11**Preparation of Investigational New Drug (IND) application for regulatory submission

**9.12**Optimisation and manufacturing of new formulations to support label-expansion* for new patient sub-populations (e.g. infants, pregnant women)

**10. CLINICAL DEVELOPMENT - PHASE I**

*First-in-human clinical trials to determine safety and tolerability of investigational new drugs in a small group of patients or healthy volunteers, including:*

**10.1**Phase Ia single ascending dose studies to determine pharmacokinetics, pharmacodynamics, and maximum tolerated dose

**10.2**Phase IIb multiple ascending dose studies to determine the pharmacokinetics, pharmacodynamics, safety and tolerability of multiple doses

**10.3**Trials of food effect or drug-drug interactions

**11. CLINICAL DEVELOPMENT - PHASE II**

*Clinical trials to determine the efficacy, safety and therapeutic dose of investigational new drugs in a small set of human subjects (up to several hundred), including:*

**11.1**Phase IIa proof of concept studies to demonstrate clinical efficacy or biological activity

**11.2**Phase IIb dose-finding studies to determine dose with optimum biological activity with minimal adverse effects

**12. CLINICAL DEVELOPMENT - PHASE III**

*Clinical trials to support the registration of investigational new drugs or label-expansion of already registered drugs in a trial population large enough to provide statistical significance (from several hundred to several thousand)*

**12.1**Regulatory standard clinical trials to assess effectiveness of a new drug against current ‘gold standard’

**12.2**Regulatory standard clinical trials that support a formal registration for label- expansion* of an existing drug to a new disease or patient group (e.g. paediatric patients, pregnant women or HIV-positive patients)

**12.3**Regulatory standard clinical trials that support formal registration for label- expansion* of an existing drug to a new use, such as intermittent preventative therapy and pre-exposure prophylaxis

**13. CLINICAL DEVELOPMENT - BASELINE EPIDEMIOLOGY**

*Studies evaluating potential trial site populations to confirm disease incidence, prevalence or exposure risk, and which serve as the foundation for determining the optimal collection, analysis, interpretation and presentation of clinical trial data; including:*

**13.1**Epidemiological studies **directly** linked to the conduct or support of clinical trials of products in development, in order to assess or validate the epidemiology of disease, disease incidence, or health of target populations at trial sites

**13.2**Preliminary studies of morbidity and mortality at potential clinical trial sites, where these studies are directly linked to planned product trials

**13.3**Pre-trial activities designed to understand trial site conditions before the commencement of trials and to facilitate engagement

**14. CLINICAL DEVELOPMENT - UNSPECIFIED**

*Other costs required to support clinical testing of investigational new drugs as needed for regulatory approval; including:*

**14.1**Infrastructure and site development costs **directly** associated with the conduct of clinical trials for drug development in **LMICs** (e.g. refurbishment of hospital wing, vehicle purchase, generators, training and community relationship building)

**14.2**Further pharmaceutical development to generate the final clinical formulation, dosage form and other Chemistry and Manufacture Control (CMC) activities required for regulatory submission

**14.3**Compiling of all non-clinical and clinical data for submission of a New Drug Application (NDA) to regulatory authorities

**14.4**Behavioural research **prior to registration** relating to risk assessment, factors affecting adherence to protocol, and product acceptability

**14.5**Protocol development, investigator meetings, Good Clinical Practice (GCP)- monitoring, quality control, data management, analysis and reporting, establishing a Data Safety Monitoring Board (DSMB), and trial audits

**15. POST-REGISTRATION STUDIES**

*Studies relating to the detection, monitoring, evaluation, and prevention of adverse events associated with newly approved drugs so as to bridge the gap between highly controlled clinical trials intended for regulatory approval and the largely uncontrolled use of new drugs by patients. Also includes studies conducted after regulatory approval that assess drug effectiveness in the wider population or which are necessary to support product use in LMICs.*

1. **15.1**Pharmacovigilance and post-registration studies of newly registered drugs to assess adverse events, toxicology and safety
2. **15.2**Effectiveness studies and head-to-head comparator studies of newly registered drugs (versus other therapies or interventions)
3. **15.3**Cost-effectiveness studies of newly registered drugs
4. **15.4**Treatment interactions and population level studies (of newly registered products e.g., pharmaco-epidemiological and resistance studies)
5. **15.5**Behavioural research **post-registration** of new drugs relating to risk assessment, factors affecting adherence to protocol, provider compliance, and product acceptability
6. **15.6**Case history reports and assessment of long-term prophylaxis using newly registered drugs in communities in LMICs

**III. VACCINES**

*Research activities and processes necessary to develop and improve investigational vaccines specifically intended to prevent infection; including vaccine design, preclinical and clinical development and other activities essential for successful vaccine development and uptake. Please see section X for disease-specific restrictions to research activities in this category.*

**16. DISCOVERY AND PRECLINICAL**

*Research activities targeted at discovering and optimising investigational vaccines and including the processes necessary to allow a candidate vaccine to proceed to human trials; including:*

**16.1**Studies supporting novel vaccine design, including target validation & candidate optimisation

**16.2**Development of animal models to assist in vaccine design and testing

**16.3**Evaluation of vaccine technologies (e.g. adjuvants, delivery systems) to improve the immunogenicity of an identified candidate

**16.4**Preclinical safety and immunogenicity studies with candidate vaccines, including use or development of functional assays

**16.5**Preclinical animal studies, challenge models and addressing the correlation between *in vitro* models, animal models and field results

**16.6**Studies on the genetics of the immune response to selected antigens as vaccine candidates, optimisation of animal models and correlates to clinical results

**16.7**Manufacturing scale-up and consistency of manufacture, including production of Good Laboratory Practice (GLP) and Good Manufacturing Practice (GMP) batches for regulatory toxicology studies and other Chemistry and Manufacture Control (CMC) activities required to allow a candidate vaccine to proceed to human trials

**16.8**Research on safety and regulatory considerations (e.g. validation of preclinical assays to permit registration)

**16.9**Preparation of an Investigational New Drug (IND) application for regulatory submission

**16.10**Optimisation of vaccine candidates for global use (cheaper, more stable, ease of administration, addition of LMIC-specific strains)

**17. CLINICAL DEVELOPMENT - PHASE I**

*First-in-human clinical trials to determine the safety of investigational new vaccines for the first time in human subjects (up to one hundred) including:*

**17.1**Phase Ia studies assessing safety, dosing, and immunogenicity in human volunteers

**17.2**Phase Ib studies assessing safety, dosing, and immunogenicity in clinically exposed or high-risk populations

**18. CLINICAL DEVELOPMENT - PHASE II**

*Clinical trials to continue to determine the efficacy and safety of investigational new vaccines in a small set of human subjects (typically several hundred), including:*

**18.1**Phase IIa challenge studies

**18.2**Phase IIb safety and preliminary efficacy studies in exposed populations or those at high-risk of infection

**19. CLINICAL DEVELOPMENT - PHASE III**

*Clinical trials to demonstrate the safety and efficacy in a larger human subject population (from several hundred to several thousand) and support the registration of investigational new vaccines, including:*

**19.1** Phase III expanded efficacy, effectiveness and safety studies required for registration purposes, including implementation, retention and follow-up of volunteers

**20. CLINICAL DEVELOPMENT - BASELINE EPIDEMIOLOGY**

*Studies evaluating potential trial site populations to confirm disease incidence, prevalence or exposure risk, and which serve as the foundation for determining the optimal collection, analysis, interpretation and presentation of clinical trial data; including:*

**20.1**Epidemiological studies **directly** linked to the conduct or support of clinical trials of preventive vaccines in development, in order to assess or validate the epidemiology of disease, disease incidence, or health of target populations at trial sites

**20.2**Preliminary studies of morbidity and mortality at potential clinical trial sites, where these studies are directly linked to planned preventive vaccines trials

**20.3**Pre-trial activities designed to understand trial site conditions before the commencement of trials and to facilitate engagement

**21. CLINICAL DEVELOPMENT - UNSPECIFIED**

*Other costs required to support clinical testing of investigational new vaccines as needed for regulatory approval; including:*

**21.1**Infrastructure and site development costs associated with the conduct of clinical trials for vaccine development in **LMICs** (e.g. refurbishment of hospital wing, vehicle purchase, generators, training and community relationship building)

**21.2**Further biological/product development to generate the optimal clinical formulation and dosage form, and other Chemistry and Manufacture Control (CMC) activities required for regulatory submission

**21.3**Compiling all non-clinical and clinical data to obtain a Biologics License from regulatory authorities

**21.4**Behavioural research **prior to registration** relating to risk assessment, factors affecting adherence to protocol, and product acceptability

**21.5**Protocol development, investigator meetings, Good Clinical Practice (GCP) monitoring, quality control, data management, analysis and reporting, establishing a Data Safety Monitoring Board (DSMB) and trial audits

**22. POST-REGISTRATION STUDIES**

*Studies relating to the detection, monitoring, evaluation, and prevention of adverse events associated with newly approved vaccines so as to bridge the gap between highly controlled clinical trials intended for regulatory approval and the largely uncontrolled delivery of new vaccines. Also includes studies conducted after regulatory approval that assess vaccine effectiveness in the wider population or which are necessary to support product use in LMICs.*

**22.1**Pharmacovigilance and post-registration studies of newly registered preventive vaccines to assess adverse reactions, toxicology and safety

**22.2**Effectiveness studies and head-to-head comparator studies of newly registered preventive vaccines (with other therapies or interventions)

**22.3**Cost-effectiveness studies of newly registered preventive vaccines

**22.4**Treatment interactions and population level studies (of newly registered preventive vaccines e.g., pharmaco-epidemiological and resistance studies)

**22.5**Behavioural research **post-registration** of new preventive vaccines relating to risk assessment, factors affecting adherence to protocol, provider compliance, and product acceptability

**22.6**Case history reports and assessment of long-term prophylaxis using newly registered preventive vaccines in communities in LMICs

**IV. BIOLOGICS**

*Research activities and processes necessary to develop and improve investigational biological agents specifically intended to prevent or treat infection; including design, preclinical and clinical development, and other activities essential for successful development and uptake. This includes broadly neutralising monoclonal antibodies (bNAbs); polyclonal antibodies; and other bio-therapeutics such as peptide-, DNA- and RNA-based synthetic molecules. Please see section X for disease-specific restrictions to research activities in this category.*

**23. DISCOVERY AND PRECLINICAL**

*Research activities targeted at discovering and optimising investigational biologics and including the processes necessary to allow a candidate biologic to proceed to human trials; including:*

**23.1**Studies supporting novel biologic design including target validation, characterisation and selection

**23.2**Candidate screening and lead optimisation

**23.3**Development of analytical tests for assaying biologics, including the development of animal models

**23.4**Evaluation of biologic technologies (e.g. adjuvants, delivery systems) to improve the immunogenicity or delivery of an identified candidate

**23.5**Biologic pharmacokinetic, toxicity and metabolism studies *in vitro* and in animal models, including bioavailability, adsorption, metabolism, and excretion (ADME) studies

**23.6**Preclinical safety and immunogenicity studies with candidate biologics, including use or development of functional assays

**23.7**Preclinical animal studies, challenge models, and studies addressing the correlation between *in vitro* models, animal models and field results necessary to satisfy Investigational New Drug (IND) requirements

**23.8**Process development and scale-up manufacture, including production of Good Laboratory Practice (GLP) and Good Manufacturing Practice (GMP) batches for regulatory toxicology studies and other Chemistry and Manufacture Control (CMC) activities required to allow a candidate biologic to proceed to human trials

**23.9**Research on safety and regulatory considerations (e.g. validation of preclinical assays to permit registration)

**23.10**Preparation of an Investigational New Drug (IND) application for regulatory submission

**23.11**Optimisation of biologic candidates for global use (cheaper, more stable, ease of administration, addition of LMIC-specific targets)

**24. CLINICAL DEVELOPMENT - PHASE I**

*First-in-human clinical trials to determine the safety and tolerability of investigational new biologics in a small group of patients or healthy volunteers, including:*

**24.1**Phase Ia studies assessing safety, dosing and immunogenicity in human volunteers; including, pharmacokinetic dynamics and tolerance in healthy volunteers.

**24.2**Phase Ib studies assessing safety, dosing and immunogenicity in clinically exposed or high-risk populations

**25. CLINICAL DEVELOPMENT - PHASE II**

*Clinical trials to determine the efficacy, safety and therapeutic dose of investigational new biologics in a small set of human subjects (up to several hundred), including:*

**25.1**Phase IIa challenge studies

**25.2**Phase IIb safety and preliminary efficacy studies in exposed populations or those at high-risk of infection

**26. CLINICAL DEVELOPMENT - PHASE III**

*Clinical trials to support the registration of investigational new drugs or label-expansion of already registered drugs in a trial population large enough to provide statistical (typically several hundred), including:*

**26.1** Phase III expanded efficacy, effectiveness and safety studies required for registration purposes, including implementation, retention and follow-up of volunteers

**27. CLINICAL DEVELOPMENT - BASELINE EPIDEMIOLOGY**

*Studies evaluating potential trial site populations to confirm disease incidence, prevalence or exposure risk, and which serve as the foundation for determining the optimal collection, analysis, interpretation and presentation of clinical trial data; including:*

**27.1**Epidemiological studies **directly** linked to the conduct or support of clinical trials of biologics in development, in order to assess or validate the epidemiology of disease, disease incidence, or health of target populations at trial sites

**27.2**Preliminary studies of morbidity and mortality at potential clinical trial sites, where these studies are directly linked to planned product trials

**27.3**Pre-trial activities designed to understand trial site conditions before the commencement of trials and to facilitate engagement

**28. CLINICAL DEVELOPMENT - UNSPECIFIED**

*Other costs required to support clinical testing of investigational new biologics as needed for regulatory approval; including:*

**28.1**Infrastructure and site development costs **directly** associated with the conduct of clinical trials for biologic development in **LMICs** (e.g. refurbishment of hospital wing, vehicle purchase, generators, training and community relationship building)

**28.2**Further product development to generate the final clinical formulation, dosage form and other Chemistry and Manufacture Control (CMC) activities required for regulatory submission

**28.3**Compiling of all non-clinical and clinical data to obtain a Biologics License from regulatory authorities

**28.4**Behavioural research **prior to registration** relating to risk assessment, factors affecting adherence to protocol, and product acceptability

**28.5**Protocol development, investigator meetings, Good Clinical Practice (GCP)- monitoring, quality control, data management, analysis and reporting, establishing a Data Safety Monitoring Board (DSMB), and trial audits

**29. POST-REGISTRATION STUDIES**

*Studies relating to the detection, monitoring, evaluation, and prevention of adverse events associated with newly approved biologics so as to bridge the gap between highly controlled clinical trials intended for regulatory approval and the largely uncontrolled use of new biologics by patients. Also includes studies conducted after regulatory approval that assess biologic effectiveness in the wider population or which are necessary to support product use in LMICs.*

**29.1**Studies conducted after regulatory approval that assess biologic effectiveness in the wider population or which are necessary to support product use in LMICs

**29.2**Pharmacovigilance and post-registration studies of newly registered biologics to assess adverse reactions, toxicology and safety

**29.3**Effectiveness studies and head-to-head comparator studies of newly registered biologics (with other therapies or interventions)

**29.4**Cost-effectiveness studies of newly registered biologics

**29.5**Treatment interactions and population level studies (of newly registered biologics e.g., pharmaco-epidemiological and resistance studies)

**29.6**Behavioural research **post-registration** of new biologics relating to risk assessment, factors affecting adherence to protocol, provider compliance, and product acceptability

**29.7**Case history reports and assessment of long-term prophylaxis using newly registered biologics in communities in LMICs

**V. DIAGNOSTICS**

*Research activities and processes necessary to develop, optimise, and validate diagnostic tests for use in resource-limited settings (cheaper, faster, more reliable, ease of use in the field); including discovery and design, preclinical and clinical evaluation, and other activities essential for successful deployment for public health use. Please see section X for disease-specific restrictions to research activities in this category.*

**30. DISCOVERY AND PRECLINICAL**

*Research activities targeted at discovering and optimising* ***low-cost****, stable, easy-to-use diagnostics for neglected diseases including the processes necessary to allow a potential product to proceed to clinical evaluation; including:*

**30.1**Validation, characterisation, and selection of targets suitable for diagnostic use

**30.2**Validation of new diagnostic markers or biomarkers

**30.3**Development and testing of **low-cost**, stable, easy-to-use diagnostic tests (e.g. simpler microscopy, improved sample collection/preparation, cheaper ELISA assays), including manufacturing design

**30.4**New or improved diagnostics for disease staging and therapy decisions

**30.5**New or improved diagnostic tools to identify resistant pathogens

**30.6**New or improved diagnostics to identify specific target populations

**30.7**Tailoring diagnostic tools for LMIC use, including improved point-of-care tests (rapid test), local laboratory test, reference laboratory tests and central laboratory tests

**30.8**Creation of reference material banks

**31. CLINICAL EVALUATION**

*Activities and processes associated with clinical evaluation of investigational diagnostic tools so as to demonstrate sensitivity and specificity in human subjects, together with other costs required to support such clinical trials; including:*

**31.1**Clinical efficacy trials

**31.2**Small-scale testing in humans to establish sensitivity and specificity and utility

**31.3**Technical evaluation of tests and studies evaluating product performance

**31.4**Establishment of product specifications, kit development and quality assurance

**31.5**Submission of relevant data to regulatory authorities for approval

**31.6**Assessment & validation of trial sites to carry out product trials

**31.7**Infrastructure and site development costs **directly** associated with the conduct of clinical trials for diagnostic development in **LMICs** (e.g. refurbishment of hospital wing, vehicle purchase, generators, training and community relationship building)

**32. OPERATIONAL RESEARCH FOR DIAGNOSTICS**

*Operational procedures and implementation activities associated with novel diagnostic tools, which are necessary to support World Health Organization recommendations for global public health use including:*

**32.1**Larger-scale demonstration studies (assessing specificity, sensitivity and utility of the diagnostic test in LMICs)

**32.2**Cost-effectiveness studies assessing the diagnostic test

**32.3**Identification of pitfalls of the technology and studies of safety measures needed to support the technology

**32.4**Studies to determine at what level of the health care system the technology is applicable (e.g. reference labs, regional labs)

**32.5**Identification of training needs

**32.6**Collecting evidence for expanding the use of a diagnostic tool in different countries

**32.7**Development of equipment and customer support documents

**32.8**Head-to-head comparator studies (with current gold standard) and in the context of existing diagnostic algorithms

**32.9**Behavioural research relating to risk assessment, factors affecting diagnostics use, and user acceptability (patient and provider)

**32.10**Epidemiological studies to assess or validate the epidemiology of disease, disease incidence or health of target populations at potential trial sites, and which are **directly** linked to clinical trials of a new diagnostic

**VI. VECTOR CONTROL PRODUCTS**

*Research and development activities and processes necessary to develop and improve vector control approaches intended to prevent infection and block transmission of a neglected disease from vector and/or animal reservoirs to human; including novel chemical vector control products, biological vector control products and reservoir targeted vaccines.*

**33. CHEMICAL VECTOR CONTROL PRODUCTS**

*This product category* ***ONLY*** *includes chemical active ingredients and formulations intended for global public health use and which specifically aim to inhibit, kill and/or repel indoor and outdoor vectors associated with neglected disease transmission. This includes new insecticides and formulations in LLINs/IRS; insecticide-based bait and traps; spatial repellents; systemic insecticides and endectocides; and chemical larvicides. Predation measures, habitat control and infrastructure measures are* ***EXCLUDED*** *from the G-FINDER scope.*

**33.1 Primary and secondary screening and optimisation**

*Laboratory-based design, synthesis and testing of potential insecticides, chemical larvicides, molluscides, trypanocides etc. and generation of data sufficient to allow developers to proceed field testing, including:*

33.1.1 Primary and secondary screens (e.g. in vitro and in vivo screens, chemical screens, whole insect screens)

33.1.2 Target validation and characterisation

33.1.3 Lead optimisation, synthesis optimisation

**33.2 Development**

*Pre-registration activities and processes associated with clinical testing of investigational chemical vector control products so as to generate data sufficient to allow developers to proceed to product roll- out & dissemination and including other costs required to support such clinical trials.*

33.2.1  Small-scale efficacy studies, residue plots and field studies necessary for product optimisation and registration

33.2.2  Acute and long-term toxicology and ecotoxicology studies

33.2.3  Metabolic and residual fate studies, crop residue and exposure data

33.2.4  Environmental assessment and environmental chemistry data

33.2.5  Generation of hazard data in humans, domestic animals and non-target plants and animals

33.2.6  Compiling of all laboratory and field data necessary for submission to regulatory authorities

33.2.7  Behavioural research conducted **pre-registration** relating to risk assessment, factors affecting adherence to protocol, and product acceptability

33.2.8  Manufacturing process development, formulation and scale-up

**33.3 PQ listing and regulatory approval**

*PQ assessment processes and post-registration research activities that comprise entomological, quality, safety and epidemiological evaluation (where appropriate) and development of specifications required for application of insecticide products for use in international public health programmes, including:*

33.3.1  PQ assessment of laboratory studies (e.g. determining intrinsic insecticidal activity, diagnostic concentration, irritant or excito-repellent properties, cross-resistance to other insecticides, efficacy and residual activity on relevant substrates)

33.3.2  PQ assessment of small-scale field trials (e.g. efficacy and persistence under different ecological settings, dosage of application, handling and application, perceived side- effects)

33.3.3  PQ assessment of large-scale field trials (e.g. community level efficacy and residual activity, operational and community acceptance)

- - 1. Assessment & validation of trial sites **directly** linked to product trials

33.3.5  Infrastructure and site development costs associated with the conduct of field trials for pesticide development in **LMICs** (e.g. refurbishment of hospital wing, vehicle purchase, generators, training and community relationship building)

33.3.6  Behavioural research conducted **post-registration** relating to risk assessment, factors affecting adherence to protocol, provider compliance and product acceptability

33.3.7  Studies that confirm efficacy, improve product uptake or confirm safety (e.g. studies to measure impact, usage levels, contamination potential or storage and disposal needs)

33.3.8  Surveillance studies directly linked to the conduct of field trials for vector control products; including studies that determine prevalence, track distribution, abundance, or significant habits of target vectors or the vector-borne pathogen

**34. BIOLOGICAL VECTOR CONTROL PRODUCTS†**

*This product category* ***ONLY*** *includes research and development of innovative biological control interventions that specifically aim to kill or control vectors associated with transmitting neglected diseases (e.g. microbial/bacteriological larvicides, sterilisation techniques, and genetic modification measures).*

*Biological control interventions comprise the use of natural enemies or "engineered" products to manage vector populations either through the introduction of natural parasites, pathogens or predators of the target, or via the introduction of modified vector species to compete with natural sources.*

*Predation measures, habitat control and infrastructure measures are* ***EXCLUDED*** *from the G-FINDER scope.*

**34.1 Phase I**

*Laboratory studies of novel biological vector control techniques*

34.1.1  Development of intervention concept and target product profile (TPP) that also specifies the intended product claim (e.g. target vector, entomological effect etc.)

34.1.2  Molecular, genotypic, physiological and behavioural characteristics research in genetically modified vectors

34.1.3  Activities related to generating transgenic vector lines, checking stability of the transgene and its phenotype and studies related to the rate of spread of a transgene in laboratory cage populations

- - 1. Ecological modelling to assess environmental risk

34.1.5  Quality control to ensure new biological materials are well characterised, stable and detectable

34.1.6  Phenotypic evaluation research of transgenic endemic strains, including testing for adverse effects on target or non-target species

- - 1. Laboratory assays to establish mechanism of action
    2. Small-scale laboratory studies for efficacy and safety

34.1.9  Laboratory-based studies on efficacy and safety in larger population cages

34.1.10Establishment of standard operating procedures for genetically modified vector production and release

34.1.11  Activities related to site preparation and hazard containment (risk analysis and risk management)

- - 1. Activities related to data analysis as required by regulators

34.1.13  Modelling of expected cost of protection per person

**34.2 Phase II**

*Semi-field tests or small-scale field trials (in physical or ecological confinement) to assess the entomological efficacy of the approach‡*

34.2.1  Physically confined (large cage, greenhouse or screen-house type facility that simulates the disease-endemic setting) field trials or semi-field tests to assess entomological efficacy (biological and functional)

34.2.2  Ecologically confined (geographic/spatial and/or climatic isolation) small-scale field trials to assess entomological efficacy (biological and functional)

- - 1. Ecological modelling to assess environmental risk

34.2.4  Compiling all entomological and epidemiological efficacy data as required by regulators

34.2.5  Activities related to site preparation and hazard containment (risk analysis and risk management)

- - 1. Initial cost analysis of prototype or approach

34.2.7  Continued monitoring of molecular quality control

**34.3 Phase III**

*Large-scale staged field trials to assess the epidemiological efficacy of the approach§*

34.3.1  Staged, open, large-scale randomised control trials to determine epidemiological efficacy (e.g., reduced disease prevalence, population suppression of target vector)

- - 1. Ecological modelling to assess environmental risk

34.3.3  Trial site selection and preparation

34.3.4  Baseline studies such as ovitrap surveillance

- - 1. Rearing and sorting of genetically modified vectors

34.3.6  Continued monitoring of molecular quality control

- - 1. Activities related to data management and statistical analysis

34.3.8  Projection of cost per person protected and cost-efficacy of prototype or approach

**34.4 Phase IV**

*Studies, in real-world conditions, that validate the effectiveness of a newly-developed biological vector control product, or post-implementation surveillance of safety and quality*

- - 1. Pilot implementation studies

34.4.2  Post-implementation studies to validate feasibility, acceptability and cost-effectiveness

34.4.3  Post-implementation surveillance studies to measure mechanism of distribution, molecular quality control, efficacy and safety (including ecological safety) that are NOT part of routine disease or demographic surveillance activities

**35. RESERVOIR TARGETED VACCINES**

*This product category* ***ONLY*** *includes research and development of veterinary vaccines specifically designed to prevent animal to human transmission of neglected diseases. Vaccines developed and used solely for veterinary purposes are excluded from this product category.*

**35.1 Discovery and preclinical**

*Research activities targeted at discovering and optimising investigational vaccines and including the processes necessary to allow a candidate vaccine to proceed to animal trials; including:*

35.1.1  Studies supporting novel vaccine design, including target validation and candidate optimisation

- - 1. Development of animal models to assist in vaccine design and testing

35.1.3  Evaluation of vaccine technologies (e.g. adjuvants, delivery systems) to improve the immunogenicity of an identified candidate

35.1.4  Preclinical safety and immunogenicity studies with candidate vaccines, including use or development of functional assays

35.1.5  Preclinical animal studies, challenge models and addressing the correlation between *in vitro* models, animal models and field results

35.1.6  Studies on the genetics of the immune response to selected antigens as vaccine candidates, optimisation of animal models and correlates to clinical results

35.1.7  Manufacturing scale-up and consistency of manufacture, including production of Good Laboratory Practice (GLP) and Good Manufacturing Practice (GMP) batches for regulatory toxicology studies and other Chemistry and Manufacture Control (CMC) activities required to allow a candidate vaccine to proceed to human trials

35.1.8  Research on safety and regulatory considerations (e.g. validation of preclinical assays to permit registration)

35.1.9  Preparation of a Veterinary Biological Product License application for regulatory submission

35.1.10  Optimisation of vaccine candidates for global use (cheaper, more stable, ease of administration, addition of LMIC-specific strains)

**35.2 Clinical development**

*Activities and processes associated with clinical testing of investigational vaccines so as to demonstrate safety, immunogenicity and efficacy in animals including animal to human transmission (as needed for regulatory approval), together with other costs required to support such clinical trials, including:*

35.2.1  Phase Ia studies assessing safety, dosing, and immunogenicity in animals; Phase Ib studies assessing safety, dosing, and immunogenicity in clinically exposed or high-risk animal populations

35.2.2  Phase IIa challenge studies; Phase II safety and preliminary efficacy studies in exposed animal populations or those at high-risk of infection

35.2.3  Phase III expanded efficacy, effectiveness and safety studies required for registration purposes

35.2.4  Infrastructure and site development costs associated with the conduct of clinical trials for vaccine development in **LMICs** (e.g. vehicle purchase, generators, training and community relationship building)

35.2.5  Further biological/product development to generate the optimal clinical formulation and dosage form, and other Chemistry and Manufacture Control (CMC) activities required for regulatory submission

35.2.6  Compiling all non-clinical and clinical data to obtain a Biologics License from regulatory authorities

35.2.7  Behavioural research **during clinical trials** relating to risk assessment, factors affecting adherence to protocol, and product acceptability

35.2.8  Protocol development, investigator meetings, Good Clinical Practice (GCP) monitoring, quality control, data management, analysis and reporting, establishing a Data Safety Monitoring Board (DSMB) and trial audits

**35.3 Phase IV/pharmacovigilance**

*Studies relating to the detection, monitoring, evaluation, and prevention of adverse events associated with newly approved vaccines so as to bridge the gap between highly controlled clinical trials intended for regulatory approval and the largely uncontrolled delivery of new vaccines. Also includes studies conducted after regulatory approval that assess vaccine effectiveness in real world settings or which are necessary to support product use in LMICs.*

35.3.1  Pharmacovigilance and post-registration studies of newly registered preventive vaccines to assess adverse reactions, toxicology and safety

35.3.2  Effectiveness studies and head-to-head comparator studies of newly registered preventive vaccines (with other therapies or interventions)

- - 1. Cost-effectiveness studies of newly registered preventive vaccines

35.3.4  Treatment interactions and population level studies (of newly registered preventive vaccines, e.g. pharmaco-epidemiological and resistance studies)

35.3.5  Behavioural research **post-registration** of new preventive vaccines relating to risk assessment, factors affecting adherence to protocol, provider compliance, and product acceptability

35.3.6 Case history reports and assessment of long-term prophylaxis using newly registered preventive vaccines in LMICs

**35.4 Baseline epidemiology**

*Studies evaluating potential trial site animal populations to confirm disease incidence, prevalence or exposure risk, and which serve as the foundation for determining the optimal collection, analysis, interpretation and presentation of clinical trial data, including*

35.4.1  Epidemiological studies **directly** linked to the conduct or support of clinical trials of vaccines in development, in order to assess or validate the epidemiological impact on disease, disease incidence, or health of target animal populations at trial sites

35.4.2  Preliminary studies of morbidity and mortality at potential clinical trial sites, where these studies are directly linked to planned vaccines trials

35.4.3  Pre-trial activities designed to understand trial site conditions before the commencement of trials and to facilitate engagement
